# Bis{2-meth­oxy-6-[(3-methoxy­prop­yl)imino­meth­yl]phenolato-κ^2^ *N*,*O* ^1^}copper(II)

**DOI:** 10.1107/S1600536808035289

**Published:** 2008-11-08

**Authors:** Amitabha Datta, Jui-Hsien Huang, Hon Man Lee

**Affiliations:** a National Changhua University of Education, Department of Chemistry, Changhua 50058, Taiwan

## Abstract

The title complex, [Cu(C_12_H_16_NO_3_)_2_], adopts a distorted square-planar coordination geometry with the Cu^II^ ion situated on a crystallographic inversion center. The two Schiff base ligands are coordinated in a *trans* fashion. In the crystal structure, non-classical inter­molecular C—H⋯O hydrogen bonds involving the ether O atoms link the Schiff base mol­ecules into a two-dimensional network parallel to (101).

## Related literature

For similar copper(II) structures with Schiff base ligands: see: Akitsu & Einaga (2004 ▶); Bluhm *et al.* (2003 ▶); Castiñeiras *et al.* (1990 ▶); Costamagna *et al.* (1998 ▶); King *et al.* (1973 ▶); Lacroix *et al.* (2004 ▶); Zhang *et al.* (2001 ▶).
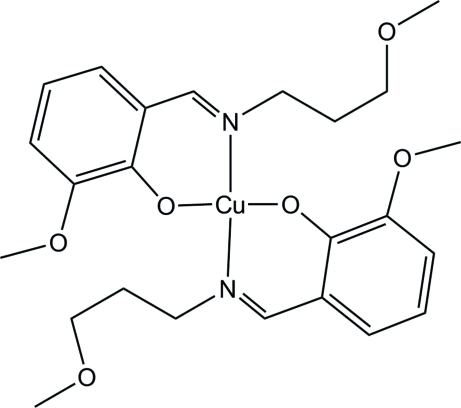


## Experimental

### 

#### Crystal data

[Cu(C_12_H_16_NO_3_)_2_]
*M* *_r_* = 508.06
Monoclinic, 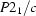

*a* = 11.2189 (9) Å
*b* = 10.7004 (8) Å
*c* = 9.5002 (7) Å
β = 96.912 (1)°
*V* = 1132.18 (15) Å^3^
*Z* = 2
Mo *K*α radiation
μ = 1.01 mm^−1^
*T* = 100 (2) K
0.50 × 0.50 × 0.40 mm

#### Data collection

Bruker SMART APEXII diffractometer
Absorption correction: multi-scan (*SADABS*; Sheldrick, 1996 ▶) *T*_min_ = 0.614, *T*_max_ = 0.668
6343 measured reflections
2298 independent reflections
2065 reflections with *I* > 2σ(*I*)
*R*_int_ = 0.032

#### Refinement

*R*[*F* ^2^ > 2σ(*F* ^2^)] = 0.027
*wR*(*F* ^2^) = 0.079
*S* = 1.09
2298 reflections
153 parameters
H-atom parameters constrained
Δρ_max_ = 0.31 e Å^−3^
Δρ_min_ = −0.37 e Å^−3^

### 

Data collection: *APEX2* (Bruker, 2004 ▶); cell refinement: *APEX2* and *SAINT* (Bruker, 2004 ▶); data reduction: *SAINT*; program(s) used to solve structure: *SHELXTL* (Sheldrick, 2008 ▶); program(s) used to refine structure: *SHELXTL*; molecular graphics: *SHELXTL*; software used to prepare material for publication: *SHELXTL*.

## Supplementary Material

Crystal structure: contains datablocks I, global. DOI: 10.1107/S1600536808035289/lh2719sup1.cif

Structure factors: contains datablocks I. DOI: 10.1107/S1600536808035289/lh2719Isup2.hkl

Additional supplementary materials:  crystallographic information; 3D view; checkCIF report

## Figures and Tables

**Table 1:** Hydrogen-bond geometry (Å, °)

*D*—H⋯*A*	*D*—H	H⋯*A*	*D*⋯*A*	*D*—H⋯*A*
C8—H8*B*⋯O3^i^	0.98	2.58	3.476 (2)	151
C9—H9*A*⋯O1^ii^	0.99	2.31	2.782 (2)	108
C9—H9*B*⋯O3	0.99	2.55	2.918 (2)	102

Symmetry codes: (i) 
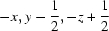
; (ii) 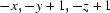.

**Fig. 1.**
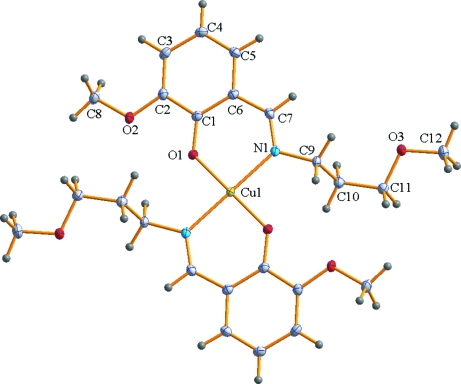
The structure of the title complex, showing 50% displacement ellipsoids for non-H atoms. The H atoms are dipicted by circles of an arbitrary radius. The unlabelled atoms are related to the labelled ones by -x, 1 - y, 1 - z.

**Fig. 2.**
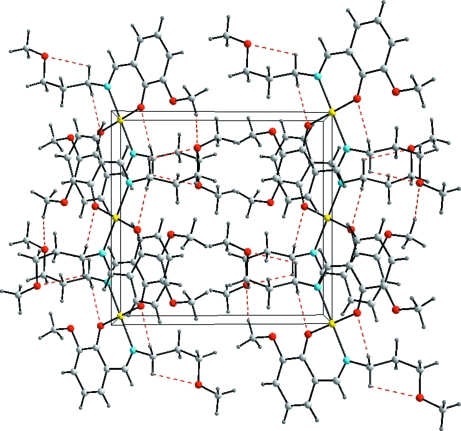
A packing diagram of the title compound along the c axis. Hyrogen bonds are shown as dashed lines.

## supplementary crystallographic information

### Comment

The Schiff base (*E*)-2-methoxy-6-[(3-methoxypropyl)iminomethyl]phenol reacts with copper(II) nitrate in methanol to form the title complex. In situ deprotonation of the phenolic hydrogen occurred leading to formation of the O/N-bidentate ligand. The title complex consists of two bidentate ligands coordinating in a *trans* fashion. It adopts a square-planar coordination geometry with the Cu atom located on a crystallographic inversion center. Schiff base Cu(II) complexes similar to the title complex have been reported in the literature (Akitsu & Einaga, 2004; Bluhm *et al.*, 2003; Castiñeiras *et al.*, 1990; Costamagna *et al.*, 1998; King *et al.*, 1973; Lacroix *et al.*, 2004; Zhang *et al.*, 2001).

Both intramolecular and intermolecular non-classical H-bonds of the type C-H···O exist (Table 1). The intermolecular H-bonds link the complex into a two-dimensional network.

### Experimental

Synthesis of (*E*)-2-methoxy-6-((3-methoxypropylimino)methyl)phenol: The compound was synthesized by the condensation reaction between O-vaniline and NH_2_(CH_2_)_3_OMe in methanol. After complete removal of the solvent, the resulting yellow liquid was used without purification.

Synthesis of the title complex: A methanolic solution of Cu(NO_3_)_2_ (1 mmol, 188 mg) and (*E*)-2-methoxy-6-((3-methoxypropylimino)methyl)phenol (2 mmol, 446 mg) was stirred for 30 min. The solution was then kept for 7 days to yield crystals suitable for X-ray diffraction study.

### Refinement

All the H atoms were positioned geometrically and refined as riding atoms, with C_aryl_—H = 0.95, C_methyl_—H = 0.98, C_methylene_—H = 0.99, C_methine_—H = 0.95 Å while *U*_iso_(H) = 1.5*U*_eq_(C) for the methyl H atoms and *U*_iso_(H) = 1.2*U*_eq_(C) for all the other H atoms.

### Crystal data

**Table:**

[Cu(C_12_H_16_N_1_O_3_)_2_]	*F*_000_ = 534
*M**_r_* = 508.06	*D*_x_ = 1.490 Mg m^−^^3^
Monoclinic, *P*2_1_/*c*	Mo *K*α radiation λ = 0.71073 Å
Hall symbol: -P 2ybc	Cell parameters from 3703 reflections
*a* = 11.2189 (9) Å	θ = 2.6–26.4º
*b* = 10.7004 (8) Å	µ = 1.01 mm^−^^1^
*c* = 9.5002 (7) Å	*T* = 100 (2) K
β = 96.9120 (10)º	Block, black
*V* = 1132.18 (15) Å^3^	0.50 × 0.50 × 0.40 mm
*Z* = 2	

### Data collection

**Table:**

Bruker SMART APEXII diffractometer	2065 reflections with *I* > 2σ(*I*)
Monochromator: graphite	*R*_int_ = 0.032
*T* = 100(2) K	θ_max_ = 26.4º
ω scans	θ_min_ = 2.6º
Absorption correction: multi-scan(SADABS; Sheldrick, 1996)	*h* = −13→7
*T*_min_ = 0.614, *T*_max_ = 0.668	*k* = −13→12
6343 measured reflections	*l* = −11→11
2298 independent reflections	

### Refinement

**Table:**

Refinement on *F*^2^	Secondary atom site location: difference Fourier map
Least-squares matrix: full	Hydrogen site location: inferred from neighbouring sites
*R*[*F*^2^ > 2σ(*F*^2^)] = 0.027	H-atom parameters constrained
*wR*(*F*^2^) = 0.079	*w* = 1/[σ^2^(*F*_o_^2^) + (0.0461*P*)^2^ + 0.0531*P*] where *P* = (*F*_o_^2^ + 2*F*_c_^2^)/3
*S* = 1.09	(Δ/σ)_max_ < 0.001
2298 reflections	Δρ_max_ = 0.31 e Å^−^^3^
153 parameters	Δρ_min_ = −0.37 e Å^−^^3^
Primary atom site location: structure-invariant direct methods	Extinction correction: none

### Special details

**Table:**

Geometry. All e.s.d.'s (except the e.s.d. in the dihedral angle between two l.s. planes) are estimated using the full covariance matrix. The cell e.s.d.'s are taken into account individually in the estimation of e.s.d.'s in distances, angles and torsion angles; correlations between e.s.d.'s in cell parameters are only used when they are defined by crystal symmetry. An approximate (isotropic) treatment of cell e.s.d.'s is used for estimating e.s.d.'s involving l.s. planes.
Refinement. Refinement of *F*^2^ against ALL reflections. The weighted *R*-factor *wR* and goodness of fit *S* are based on *F*^2^, conventional *R*-factors *R* are based on *F*, with *F* set to zero for negative *F*^2^. The threshold expression of *F*^2^ > σ(*F*^2^) is used only for calculating *R*-factors(gt) *etc*. and is not relevant to the choice of reflections for refinement. *R*-factors based on *F*^2^ are statistically about twice as large as those based on *F*, and *R*- factors based on ALL data will be even larger.

### Fractional atomic coordinates and isotropic or equivalent isotropic displacement parameters (Å^2^)

**Table:**

	*x*	*y*	*z*	*U*_iso_*/*U*_eq_	
Cu1	0.0000	0.5000	0.5000	0.01161 (11)	
N1	0.06486 (12)	0.67268 (12)	0.54175 (13)	0.0124 (3)	
O1	−0.10464 (10)	0.55690 (11)	0.34077 (12)	0.0157 (3)	
O2	−0.26520 (10)	0.57869 (11)	0.11946 (12)	0.0168 (3)	
O3	0.38383 (10)	0.83717 (11)	0.73181 (13)	0.0203 (3)	
C1	−0.12843 (14)	0.67057 (15)	0.29658 (16)	0.0123 (3)	
C2	−0.21677 (14)	0.68788 (15)	0.17655 (16)	0.0133 (3)	
C3	−0.24800 (15)	0.80571 (16)	0.12650 (17)	0.0146 (3)	
H3	−0.3084	0.8153	0.0481	0.018*	
C4	−0.19103 (15)	0.91184 (16)	0.19081 (17)	0.0160 (4)	
H4	−0.2131	0.9930	0.1564	0.019*	
C5	−0.10354 (15)	0.89779 (15)	0.30333 (17)	0.0146 (3)	
H5	−0.0639	0.9695	0.3453	0.018*	
C6	−0.07147 (15)	0.77820 (15)	0.35771 (16)	0.0128 (3)	
C7	0.02158 (15)	0.77112 (16)	0.47516 (16)	0.0134 (3)	
H7	0.0557	0.8486	0.5081	0.016*	
C8	−0.35395 (15)	0.59005 (17)	−0.00057 (17)	0.0185 (4)	
H8A	−0.4248	0.6325	0.0275	0.028*	
H8B	−0.3767	0.5067	−0.0372	0.028*	
H8C	−0.3214	0.6388	−0.0744	0.028*	
C9	0.16306 (14)	0.69566 (15)	0.65644 (16)	0.0136 (3)	
H9A	0.1512	0.6429	0.7392	0.016*	
H9B	0.1613	0.7842	0.6862	0.016*	
C10	0.28466 (15)	0.66641 (16)	0.60836 (17)	0.0166 (4)	
H10A	0.2902	0.5756	0.5904	0.020*	
H10B	0.2919	0.7107	0.5183	0.020*	
C11	0.38687 (15)	0.70517 (15)	0.71811 (18)	0.0161 (4)	
H11A	0.3781	0.6652	0.8103	0.019*	
H11B	0.4645	0.6787	0.6881	0.019*	
C12	0.47976 (15)	0.88352 (17)	0.82805 (18)	0.0214 (4)	
H12A	0.4776	0.8444	0.9210	0.032*	
H12B	0.4719	0.9743	0.8369	0.032*	
H12C	0.5562	0.8639	0.7929	0.032*	

### Atomic displacement parameters (Å^2^)

**Table:**

	*U*^11^	*U*^22^	*U*^33^	*U*^12^	*U*^13^	*U*^23^
Cu1	0.01075 (17)	0.01035 (17)	0.01270 (16)	−0.00026 (10)	−0.00282 (11)	0.00036 (10)
N1	0.0102 (7)	0.0140 (7)	0.0128 (6)	−0.0004 (6)	0.0000 (6)	−0.0016 (6)
O1	0.0166 (6)	0.0117 (6)	0.0170 (6)	0.0001 (5)	−0.0057 (5)	0.0011 (5)
O2	0.0164 (6)	0.0158 (6)	0.0163 (6)	−0.0019 (5)	−0.0061 (5)	−0.0006 (5)
O3	0.0167 (6)	0.0128 (6)	0.0286 (7)	−0.0022 (5)	−0.0085 (5)	0.0003 (5)
C1	0.0108 (8)	0.0133 (8)	0.0133 (7)	0.0012 (7)	0.0033 (6)	0.0004 (6)
C2	0.0116 (8)	0.0149 (8)	0.0138 (7)	−0.0009 (7)	0.0027 (7)	−0.0009 (6)
C3	0.0114 (8)	0.0191 (9)	0.0130 (7)	0.0020 (7)	0.0002 (6)	0.0032 (7)
C4	0.0171 (9)	0.0135 (8)	0.0176 (8)	0.0024 (7)	0.0029 (7)	0.0030 (7)
C5	0.0165 (9)	0.0108 (8)	0.0169 (8)	−0.0004 (7)	0.0032 (7)	−0.0011 (7)
C6	0.0115 (8)	0.0141 (8)	0.0132 (8)	0.0013 (7)	0.0029 (7)	0.0003 (6)
C7	0.0136 (8)	0.0120 (8)	0.0149 (8)	−0.0014 (6)	0.0024 (7)	−0.0026 (6)
C8	0.0157 (9)	0.0219 (9)	0.0166 (8)	−0.0013 (7)	−0.0039 (7)	0.0006 (7)
C9	0.0115 (8)	0.0143 (8)	0.0140 (8)	−0.0008 (7)	−0.0025 (6)	−0.0021 (6)
C10	0.0146 (9)	0.0172 (8)	0.0176 (8)	−0.0001 (7)	0.0000 (7)	−0.0027 (7)
C11	0.0136 (8)	0.0145 (8)	0.0198 (8)	0.0005 (7)	0.0002 (7)	−0.0012 (7)
C12	0.0175 (9)	0.0200 (9)	0.0256 (9)	−0.0055 (7)	−0.0022 (8)	−0.0030 (8)

### Geometric parameters (Å, °)

**Table:**

Cu1—O1^i^	1.9000 (11)	C5—C6	1.410 (2)
Cu1—O1	1.9000 (11)	C5—H5	0.9500
Cu1—N1^i^	2.0079 (13)	C6—C7	1.435 (2)
Cu1—N1	2.0079 (13)	C7—H7	0.9500
N1—C7	1.293 (2)	C8—H8A	0.9800
N1—C9	1.474 (2)	C8—H8B	0.9800
O1—C1	1.3038 (19)	C8—H8C	0.9800
O2—C2	1.3724 (19)	C9—C10	1.522 (2)
O2—C8	1.4258 (19)	C9—H9A	0.9900
O3—C12	1.415 (2)	C9—H9B	0.9900
O3—C11	1.419 (2)	C10—C11	1.512 (2)
C1—C6	1.408 (2)	C10—H10A	0.9900
C1—C2	1.430 (2)	C10—H10B	0.9900
C2—C3	1.378 (2)	C11—H11A	0.9900
C3—C4	1.406 (2)	C11—H11B	0.9900
C3—H3	0.9500	C12—H12A	0.9800
C4—C5	1.370 (2)	C12—H12B	0.9800
C4—H4	0.9500	C12—H12C	0.9800
			
O1^i^—Cu1—O1	180.0	C6—C7—H7	115.9
O1^i^—Cu1—N1^i^	92.11 (5)	O2—C8—H8A	109.5
O1—Cu1—N1^i^	87.89 (5)	O2—C8—H8B	109.5
O1^i^—Cu1—N1	87.89 (5)	H8A—C8—H8B	109.5
O1—Cu1—N1	92.11 (5)	O2—C8—H8C	109.5
N1^i^—Cu1—N1	180.00 (7)	H8A—C8—H8C	109.5
C7—N1—C9	115.41 (14)	H8B—C8—H8C	109.5
C7—N1—Cu1	123.18 (11)	N1—C9—C10	111.16 (12)
C9—N1—Cu1	121.36 (10)	N1—C9—H9A	109.4
C1—O1—Cu1	129.66 (11)	C10—C9—H9A	109.4
C2—O2—C8	116.66 (13)	N1—C9—H9B	109.4
C12—O3—C11	112.53 (13)	C10—C9—H9B	109.4
O1—C1—C6	124.41 (15)	H9A—C9—H9B	108.0
O1—C1—C2	118.23 (14)	C11—C10—C9	111.67 (13)
C6—C1—C2	117.35 (14)	C11—C10—H10A	109.3
O2—C2—C3	124.78 (15)	C9—C10—H10A	109.3
O2—C2—C1	114.10 (14)	C11—C10—H10B	109.3
C3—C2—C1	121.12 (15)	C9—C10—H10B	109.3
C2—C3—C4	120.37 (15)	H10A—C10—H10B	107.9
C2—C3—H3	119.8	O3—C11—C10	108.16 (14)
C4—C3—H3	119.8	O3—C11—H11A	110.1
C5—C4—C3	119.73 (16)	C10—C11—H11A	110.1
C5—C4—H4	120.1	O3—C11—H11B	110.1
C3—C4—H4	120.1	C10—C11—H11B	110.1
C4—C5—C6	120.85 (16)	H11A—C11—H11B	108.4
C4—C5—H5	119.6	O3—C12—H12A	109.5
C6—C5—H5	119.6	O3—C12—H12B	109.5
C1—C6—C5	120.53 (15)	H12A—C12—H12B	109.5
C1—C6—C7	121.93 (15)	O3—C12—H12C	109.5
C5—C6—C7	117.53 (15)	H12A—C12—H12C	109.5
N1—C7—C6	128.21 (16)	H12B—C12—H12C	109.5
N1—C7—H7	115.9		
			
O1^i^—Cu1—N1—C7	−173.21 (13)	C3—C4—C5—C6	−1.4 (2)
O1—Cu1—N1—C7	6.79 (13)	O1—C1—C6—C5	−179.61 (15)
O1^i^—Cu1—N1—C9	4.07 (11)	C2—C1—C6—C5	1.5 (2)
O1—Cu1—N1—C9	−175.93 (11)	O1—C1—C6—C7	1.2 (3)
N1^i^—Cu1—O1—C1	172.75 (14)	C2—C1—C6—C7	−177.67 (14)
N1—Cu1—O1—C1	−7.25 (14)	C4—C5—C6—C1	0.4 (2)
Cu1—O1—C1—C6	4.5 (2)	C4—C5—C6—C7	179.60 (14)
Cu1—O1—C1—C2	−176.60 (10)	C9—N1—C7—C6	178.39 (15)
C8—O2—C2—C3	0.2 (2)	Cu1—N1—C7—C6	−4.2 (2)
C8—O2—C2—C1	179.96 (13)	C1—C6—C7—N1	−1.1 (3)
O1—C1—C2—O2	−1.3 (2)	C5—C6—C7—N1	179.74 (16)
C6—C1—C2—O2	177.71 (13)	C7—N1—C9—C10	−101.17 (16)
O1—C1—C2—C3	178.53 (14)	Cu1—N1—C9—C10	81.35 (15)
C6—C1—C2—C3	−2.5 (2)	N1—C9—C10—C11	172.66 (13)
O2—C2—C3—C4	−178.62 (14)	C12—O3—C11—C10	−177.34 (13)
C1—C2—C3—C4	1.6 (2)	C9—C10—C11—O3	−64.55 (18)
C2—C3—C4—C5	0.4 (2)		

Symmetry codes: (i) −*x*, −*y*+1, −*z*+1.

### Hydrogen-bond geometry (Å, °)

**Table:**

*D*—H···*A*	*D*—H	H···*A*	*D*···*A*	*D*—H···*A*
C8—H8B···O3^ii^	0.98	2.58	3.476 (2)	151
C9—H9A···O1^i^	0.99	2.31	2.782 (2)	108
C9—H9B···O3	0.99	2.55	2.918 (2)	102

Symmetry codes: (ii) −*x*, *y*−1/2, −*z*+1/2; (i) −*x*, −*y*+1, −*z*+1.
